# A Novel Mutation of *VPS*33*B* Gene Associated with Incomplete Arthrogryposis-Renal Dysfunction-Cholestasis Phenotype

**DOI:** 10.1155/2020/8872294

**Published:** 2020-09-24

**Authors:** Eleni Agakidou, Charalampos Agakidis, Marios Kambouris, Nicoleta Printza, Maria Farini, Elina Vourda, Spyridon Gerou, Kosmas Sarafidis

**Affiliations:** ^1^1^st^ Department of Neonatology, Aristotle University of Thessaloniki, Ippokration General Hospital, Thessaloniki, Greece; ^2^1^st^ Department of Pediatrics, Aristotle University of Thessaloniki, Ippokration General Hospital, Thessaloniki, Greece; ^3^Division of Genetics, Department of Pathology and Laboratory Medicine, Sidra Medicine Hospital, Doha, Qatar; ^4^Analysis Medical S.A. Diagnostic—Research Clinics, Thessaloniki, Greece

## Abstract

Arthrogryposis-renal dysfunction-cholestasis (ARC) syndrome is an autosomal recessive disorder caused by mutations of the *VPS*33*B* encoding the vacuolar protein sorting 33B (VPS33B), which is involved in the intracellular protein sorting and vesicular trafficking. We report a rare case of ARC syndrome without arthrogryposis caused by a novel mutation of *VPS*33*B*. A female patient of Greek origin presented on the 14^th^ day of life with renal tubular acidosis, Fanconi syndrome, nephrogenic diabetes insipidus, and cholestasis with normal gamma-glutamyl transpeptidase, without arthrogryposis and dysmorphic features. She was born to apparently healthy, nonconsanguineous parents. Additional features included dry and scaling skin, generalized hypotonia, hypoplastic corpus callosum, neurodevelopmental delay, failure to thrive, short stature, recurrent febrile episodes with and without infections, and gastrointestinal bleeding. DNA testing revealed that the patient was homozygous for the novel c.1098_1099delTG (p.Glu367Alafs*∗*17) mutation of exon 14 of *VPS*33*B* gene (NM_018668) on chromosome 15q26.1, leading to a nonsense frameshift variant of VPS33B with premature termination of translation. Her parents were heterozygous for the same *VPS*33*B* mutation. The prognosis was predictably poor in the context of the intractable polyuria necessitating long-term parenteral fluid administration via indwelling central catheter leading to catheter-related sepsis, to which she eventually succumbed at the age of 7 months. This is the first published *VPS*33*B* mutation in an ARC patient of Greek origin. The current case adds to the spectrum of ARC-associated *VPS*33*B* mutations and provides evidence supporting the existence of incomplete ARC phenotype. Increased awareness and early genetic testing for ARC are suggested in cases with isolated cholestasis and/or renal tubular dysfunction, even in the absence of arthrogryposis.

## 1. Introduction

Arthrogryposis-renal dysfunction-cholestasis (ARC) syndrome (OMIM #208085 and #613404) is an autosomal recessive disorder caused by mutations of the *VPS*33*B* and *VIPER* genes. The *VPS*33*B* encodes the vacuolar protein sorting 33B (VPS33B) that is involved in intracellular protein sorting and vesicular trafficking [1–4]. VPS33B is ubiquitously expressed in human tissues including both liver and kidneys. Consequently, dysfunction of VPS33B may lead to disruption of cell polarization in many organs, thereby resulting in a multisystem disorder with Fanconi syndrome (FS), myoskeletal anomalies, and cholestasis with normal gamma-glutamyl transpeptidase (GGT) being the core manifestations [5, 6]. Additional features of ARC include nephrogenic diabetes insipidus (NDI), failure to thrive, anomalies of the corpus callosum with neurodevelopmental delay, ichthyosis, platelet dysfunction, recurrent infections, dysmorphic features, congenital heart disease, hypothyroidism, and keratitis. The disease is more common in Koreans, Pakistani, and Arabians, while cases in patients of South American, Turkish, and European origin (Leiden Open-Source Variation Database (LOVD), for ARC available at https://grenada.lumc.nl/LOVD2/ARC/home.php?select_db=VPS33B accessed on 4/1/2020) have also been reported [7]. Herein, we report on a female infant homozygous to a novel mutation of the *VPS*33*B* gene that presented with incomplete ARC phenotype including two of the three core manifestations, namely, FS and cholestasis, without arthrogryposis or dysmorphic features. ARC phenotype without arthrogryposis is extremely rare and may delay the diagnosis of the syndrome. Moreover, this is the first report of genetically confirmed ARC syndrome in a patient of Greek origin. Written informed consent for the publication was obtained from the parents.

## 2. Case Presentation

A 14-day-old female neonate was admitted to a tertiary neonatal intensive care unit (NICU) with dehydration and hypernatremia. She was the second-born child of apparently healthy parents without known consanguinity, who originated from the same village. Her six-year-old male brother was healthy and the family history for early infantile deaths and liver, kidney, or skeletal diseases was unremarkable. Fetal ultrasound (US) findings at 11 and 21 weeks of gestation were normal. Pregnancy was complicated by mild preeclampsia. She was born at 37 weeks of gestation via elective cesarean section, with a birth weight of 2810 g (26^th^ centile), length of 48 cm (24^th^ centile), and head circumference of 33 cm (20^th^ centile). On physical examination, the newborn was apparently normal without any dysmorphic features and was exclusively breastfed.

As shown in the timeline table (Table 1), the patient was admitted to the hospital on the 14^th^ day of life for excessive weight loss (14% of birth weight), despite adequate fluid and energy intake. On admission, she was noted to be dehydrated with increased urine output (6–15 mL per kg per hour). Laboratory tests showed hyperchloremic metabolic acidosis (base excess −11 mmol/L, bicarbonate 15.5 mmol/L, Cl 126 mmol/L) with normal anion gap and lactic acid. She was treated with intravenous fluids, sodium bicarbonate, and electrolyte supplementation titrated against serum levels. During the following weeks, she continued to have a high urine output (8–17 ml/kg/hour) requiring excessive volumes of fluid for hydration balance (300 mL/kg/day). Biochemistry showed persisting hyperchloremic metabolic acidosis, hypernatremia (158 mmol/L), conjugated bilirubinemia (total and conjugated bilirubin 99.6 *μ*mol/L and 76.1 *μ*mol/L, respectively), elevated alkaline phosphatase (ALP 871 IU/L), and normal serum levels of transaminases, GGT, lactate dehydrogenase, and creatinine phosphokinase. A 24-hour urine examination revealed normoglycemic glycosuria, decreased renal absorption of P, and increased excretion of Na, Ca, proteins, and microalbumin, which combined with the hyperchloremic metabolic acidosis were indicative of type II renal tubular acidosis with FS. At that time point, plasma and urine amino acids, blood ammonia and lactic acid, and serum K, Ca, and Mg were within normal ranges, while urine was tested negative for reducing substances. Head, heart, and abdominal US findings were normal. Management included maintenance of fluid and electrolyte equilibrium and bicarbonate administration. On the 44^th^ day of life, she developed *Klebsiella* sepsis that was successfully treated with antibiotics. Anthropometric measurements at that point revealed growth retardation (weight and length below the 3^rd^ centile, and head circumference at the 20^th^ centile) while clinical examination was notable for dry, scaling skin, without dysmorphic appearance except for high arch palate.

During hospitalization, the patient had persistent hypernatremia and polyuria (8–17 mL/kg/hour) necessitating a daily fluid intake of up to 440 mL/kg. Moreover, she developed recurrent episodes of fever without laboratory evidence of infection as well as two episodes of catheter-related sepsis caused by *Enterobacter* and *Candida albicans*, respectively. Arterial blood pressure was maintained in the normal range throughout her hospitalization, with the exception of brief hypotensive periods during the septic episodes.

Blood and serum laboratory findings during hospitalization are summarized in Table 2. Hematological indices were remarkable for fluctuating white blood cell counts and anemia necessitating a total of four red-cell transfusions and increased platelet counts decreasing to a minimum of 133 c/fl during septic episodes. Coagulation tests including prothrombin time, partial thromboplastin time, international normalized ratio, fibrinogen, and D-Dimers were normal. Hypophosphatemia and hypernatremia were also noted while blood glucose, plasma amino acids, and serum K, Mg, and Ca were normal (Table 2). Serum immunoglobulin levels and immunophenotype were within the normal range. Urine biochemistry revealed marked increases of protein, microalbumin, and glucose excretion, decreased P reabsorption, and generalized aminoaciduria, which were compatible with FS. However, excessive polyuria along with persisting hypernatremia was suggestive of diabetes insipidus. The desmopressin test did not reduce urine output thereby excluding central diabetes insipidus while indicating NDI as a factor contributing to sustained polyuria and hypernatremia. Subsequently, hydrochlorothiazide administration reduced the daily urine output by about 30%–50% supporting the diagnosis of NDI. Urea and creatinine serum levels were normal while mild elevations were observed during dehydration and/or septic episodes (Table 2).

Liver function tests showed persisting conjugated bilirubinemia, increased serum bile acids and ALP, progressive increase of transaminases, normal GGT, hypertriglyceridemia in samples obtained either before commencing lipid administration or following interruption of parenteral lipid infusion, total serum protein at the lower normal levels, and low serum albumin. Tests for viral hepatitis were negative. Thyroid function tests, blood ammonia and lactic acid, lactate dehydrogenase, creatinine phosphokinase, alpha-fetoprotein, alpha1 antitrypsin, plasma amino acids, as well as urine organic acids, carnitine, and carnitine esters were within the reference ranges. Karyotype was also normal.

US examination of the heart, liver, bile cyst and ducts, spleen, and adrenal gland was normal, while kidneys had increased echodensity with normal size, calyces, and pelvis. Head US displayed a thin corpus callosum and the hip US showed developmental immaturity without dislocation. Brain magnetic resonance imaging (MRI) revealed delayed white matter maturation, hypoplastic corpus callosum, and normal appearance of hypothalamus and hypophysis. The MRI of the liver, gallbladder, bile ducts, kidneys, and adrenal gland was normal. Brainstem auditory evoked potentials and ophthalmologic examination were also normal. Liver and renal biopsies were not performed as parents did not consent for these investigations.

### 2.1. Diagnosis

**Figure 1:**
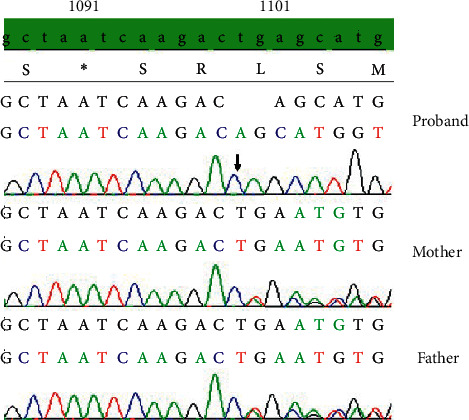
Confirmatory Sanger Sequencing of the *VPS*33*B* gene c.1098_1099delTG (p.Glu367Alafs*∗*17) mutation from the patient and her parents (NM_018668).

At 50 days of life, DNA analysis for suspected ARC was requested. Whole exome sequencing (WES) was performed using Ion AmpliSeq™ Exome RDY Panel (Thermo Fisher Scientific, Life Technologies Corp., CA) on the Next Generation Sequencing (NGS) S5/S5xl platform by Thermo Fisher Scientific. Variant interpretation (eVai, enGenome, Pavia, Italy) revealed that the patient was homozygous for the c.1098_1099delTG (p.Glu367Alafs*∗*17) mutation in exon 14 of *VPS*33*B* gene (NM_018668) (Figure 1). The mutation was confirmed by Sanger sequencing. The parental genetic investigation by Sanger sequencing showed that both parents were heterozygous for the same mutation. Other gene mutations associated with the patient's phenotype were not detected.

### 2.2. Outcome

At the age of five and a half months, the patient was still hospitalized. She continued to be growth retarded (weight and length less than the 3^rd^ centile, and head circumference at the 10^th^ centile), and under antibiotic treatment for catheter-related sepsis with *Candida albicans*. A few bowel movements of stools mixed with streaks of mucous blood resolved following enteral feeds with an amino acid formula. Neurological examination revealed marked developmental delay with generalized hypotonia, weak sucking, and swallowing difficulties. Direct bilirubin and transaminase levels progressively increased. Ongoing treatment with hydrochlorothiazide moderately impacted polyuria, decreasing the daily urine output from 15–17 mL/kg to about 8–10 mL/kg. Enteral nutrition with an amino acid formula was provided via jejunostomy and was supplemented by administration of intravenous fluids of about 200 mL/kg/day via an indwelling catheter to compensate for the excessive urine output. She was on bicarbonates (2–6 mEq/kg/day) and electrolytes titrated against serum levels, as well as on albumin administration when required. Moreover, she was receiving vitamins D, E, and K, ursodeoxycholic acid, and hydrochlorothiazide orally. Sadly, at the age of seven months, the patient succumbed to catheter-related sepsis due to *Candida albicans* that was complicated by multiorgan failure.

## 3. Discussion

The patient's main manifestations included early renal tubular dysfunction presenting with renal tubular acidosis, FS, and NDI, as well as normal GGT-cholestasis. Additional findings included severe failure to thrive, stunting, dry and scaling skin, generalized hypotonia, hypoplastic corpus callosum, neurodevelopmental delay, recurrent febrile episodes with or without evidence of infection, gastrointestinal bleeding, and platelets (PLT) with abnormal morphology and normal coagulation tests, without arthrogryposis or other dysmorphic features. Genetic tests revealed that the patient was homozygous for a novel mutation of the *VPS*33*B* gene confirming the clinical diagnosis of ARC syndrome.

Renal involvement in the current patient was manifested with FS and NDI. Tubular manifestations are attributed to defective intracellular protein trafficking leading to the disrupted epithelial polarity of the renal tubular cells which impedes directional absorption and secretion of solutes [5]. FS is a cardinal feature of the ARC caused by *VPS*33*B* mutations. The diagnosis of FS was made on grounds of hyperchloremic metabolic acidosis with a normal anion gap, hypophosphatemia, decreased phosphorus tubular reabsorption, normoglycemic glucosuria, generalized aminoaciduria, and proteinuria. Hypernatremic dehydration with high urine output up to 17 mL/kg/day and increased blood osmolality was consistent with diabetes insipidus. The unresponsiveness to desmopressin stimulation along with the normal MRI findings from hypophysis and hypothalamus excluded central diabetes insipidus suggesting NDI. NDI has been reported to occur in more than 50% of patients with ARC [8–13]. The effect of VPS33B dysfunction on collecting tubules is supported by experimental studies showing abnormal expression of membrane proteins and defective structure and function of the tight junctions of collecting duct cells in VIPAR- and VPS33B-deficient mouse [14]. Our patient required huge amounts of fluids (up to 430 mL/kg/d) to maintain fluid balance, while treatment with hydrochlorothiazide reduced the urine output by about 30% to 50% further supporting the diagnosis of NDI.

Neonatal cholestasis constitutes a diagnostic and therapeutic challenge [15]. Liver findings were compatible with intrahepatic cholestasis with normal GGT, which can be a feature of several genetic syndromes including familial intrahepatic cholestasis 1 deficiency coded by *ATP8B1*, bile salt export pump deficiency coded by *ABCB11*, farnesoid X receptor deficiency coded by *NR1H4*, myosin B protein deficiency coded by *MYO5B*, and *VPS*33*B* protein dysfunction coded by *VPS*33*B* [16]. In our case, whole exon sequencing revealed a *VPS*33*B* variance, while other gene variances associated with intrahepatic cholestasis were not identified. Liver dysfunction in ARC is attributed to a loss of the apical and basolateral hepatocyte polarity leading to the flow of bile acids and other substances into the bloodstream instead of bile ducts. In this context, experimental studies in ARC models showed that proteins that are normally localized at the apical membrane of hepatocytes were randomly located in intracellular vesicles. Consequently, they were unable to fulfill their mission, i.e., to excrete bile acids, conjugated bilirubin, ALP, cholesterol, and other substances to the bile ducts [6, 17, 18]. In accordance with experimental findings and previously reported ARC patients, our patient developed elevated serum levels of conjugated bilirubin, bile acids, ALP, and triglycerides, and mildly elevated transaminases [8, 10–12, 19–23]. Normal of GGT is a constant finding in ARC-associated intrahepatic cholestasis and can be explained by the presence of normal bile ducts. Nevertheless, rare ARC cases with increased GGT caused by *VPS*33*B* mutations have also been reported [24]. In mild cases with long-term survival, progressive liver disease may ensue leading to cirrhosis [8, 25].

Hypogranular, large PLT, and/or defective platelet function with bleeding manifestations are common in ARC patients [10–12, 20, 21, 26]. PTL dysfunction in ARC has been attributed to abnormal protein trafficking and defective maturation of multivesicular bodies in megakaryocytes secondary to VPS33B impairment, resulting in maldevelopment of the PLT *α*-granules [3, 27]. Although PLT function tests were not performed in our patient due to the high blood volume required, light microscopy findings of medium and large PLT with poor granulation are consistent with impaired development of granules as found in experimental models of *VPS*33*B* deficiency mouse [3, 27]. Several authors reported bleeding episodes of varying severity of complicating liver and renal biopsies [10–12, 22]. In the context of severe hemorrhage risk, previous authors suggested early genetic testing for confirmation of diagnosis rather than relying on renal and liver biopsies [10–12, 22, 28, 29]. The persistently increased PLT counts found in the current patient have not been reported previously in ARC patients.

Additional comorbidities in our patient described previously in ARC patients include severe growth faltering despite adequate energy intake, stunting, neurodevelopmental delay, hypoplastic corpus callosum, dry and scaling skin, recurrent febrile episodes with or without evidence of infection, and increased serum triglycerides [8, 10–13, 19, 23–25, 30].

Whole Exome Sequencing (WES) Genetic evaluation showed that the patient was homozygous for a *VPS*33*B* c.1098_1099delTG; p.Glu367Alafs*∗*17, a two-nucleotide deletion frameshift mutation in exon 14 (NM_018668) on chromosome 15q26, resulting in a stop codon after the incorporation of 17 erroneous amino acids downstream of the deletion point and premature termination of the protein sequence. This is a novel mutation not previously described in the medical and scientific literature and not present in any relevant mutation databases. No other mutations in known genes associated with the patient's phenotype were detected by WES.

Taking into account the Joint Consensus Recommendation of the American College of Medical Genetics and Genomics and the Association for Molecular Pathology for the classification of the pathogenicity of novel variants [31], this mutation is characterized as class 4 mutation with very strong evidence of pathogenicity (PVS1). Since it is a novel, previously not encountered mutation, its initial classification is a Class 3 Variant of Unknown Clinical Significance. The following evidence elevates its status to class 4 mutation with very strong evidence of pathogenicity (PVS1):

- This is a null, two-nucleotide deletion, frameshift, protein chain termination variant resulting in a stop codon after the incorporation of 17 erroneous amino acids downstream of the deletion point and a multiexon deletion (exons 14–22) of the resulting mRNA with a possible stop codon-mediated mRNA decay.
- Loss of Function (LOF) of *VPS*33*B* mutations is a well-known and widely accepted disease mechanism resulting in ARC disease.
- The LOF variant is not at the extreme 3′end of the gene.
- The mutation affects all known transcripts of the *VPS*33*B* gene.
- Multiple computational tools suggest a deleterious effect of the mutation at the protein level. All in silico mutation effect prediction software types (PolyPhen, Shift, Mutalizer, etc.) by virtue of the frameshift and stop codon encountering nature of the mutation characterize all such variants as pathogenic.
- Genomic evidence derived from targeted mutation detection for all individuals in the nuclear family (father, mother, and affected child) showed mutation to disease cosegregation for the variant with both parents being phenotypically unremarkable, heterozygous carriers of the mutation.
- The mutation is not encountered in the Genome Aggregation Database (gnomAD).
- Finally, the only issue that prevents this variant from being characterized as class 5 is that no functional assay exists to document the effect of this mutation at the mRNA or protein levels.

The reports of patients with ARC (both clinical only and also confirmed by molecular testing) with arthrogryposis as part of the clinical presentation are numerous. The molecular defects in patients with *VPS*33*B* mutations (either homozygous or compound heterozygous) show tremendous allelic heterogeneity with a full spectrum of various mutation types, including point mutations, splice-site mutations, chain-terminating mutations, and frameshift mutations followed by chain termination, deletions, duplications, without an obvious genotype to phenotype correlation. Also, a limited spectrum of clinical heterogeneity has been observed in various affected members of the same family but not usually in the three core characteristics of the disease (arthrogryposis, renal dysfunction, and cholestasis).

Our patient presented without arthrogryposis, one of the three core features of ARC. Myoskeletal abnormalities of the syndrome, such as the presence of arthrogryposis, have been attributed to neurogenic muscle atrophy [2], with evidence suggesting a role for abnormal collagen formation [32]. However, rare cases of ARC without arthrogryposis have been published. Bull et al. [23] reported a patient with ARC, homozygous for a VPS33B_971delA; K324fs*∗*10 mutation, who presented with normal levels of GGT, cholestasis, aminoaciduria, ichthyosis, partial agenesis of the corpus callosum, and sensorineural deafness, but without arthrogryposis. The nature of this mutation is very similar to the nature of the mutation for the patient described in this report. They both affect the *VPS*33*B* gene, both are homozygous, frameshift, and chain-terminating mutations, both affect the same general protein domain (amino acid positions 376 and 324, respectively), both may result in mRNA mediated decay (not proven), and both involve deletions of a limited number of bases (two bases in our case, one base in the case of the report by Bull et al. [23]). Qiu et al. [19] reported two families, a total of three patients (single patient in one family and two siblings in the other) with confirmed *VPS*33*B* mutations who had only isolated cholestasis and no additional clinical findings, without arthrogryposis. Apparently, none of the other hallmarks of ARC syndrome were present. Affected individuals in one of the families were compound heterozygous for a *VPS*33*B* point mutation and a frameshift and chain-terminating mutation (c. 1509dupG; p.Lys504Glufs*∗*), which shares certain similarities with the one in our patient (c.1098_1099delTG; p.Glu367Alafs*∗*17) as far as the frameshift and chain-terminating aspects are concerned, but the affected amino acid is located more than 130 amino acids downstream in the protein sequence. The stop-codon mRNA mediated decay of these mutations, although not proven, if present, should have a similar detrimental effect. The fact that a frame shift mutation in the report by Qiu et al. [19] is considered pathogenic and the affected amino acid is located 137 amino-acids downstream in the protein sequence than the affected amino acid in the current case, greatly supporting the pathogenic nature of the mutation in our patient too. Smith et al. [33] reported a single patient with failure to thrive, developmental delay, sensorineural hearing loss, renal loss of protein and amino acids, bilateral talipes with osteopenia, mild cholestasis, pruritus, and ichthyosis, but no arthrogryposis. The patient was compound heterozygous for two *VPS*33*B* mutations. One allele harbors a deletion c.240–577_290-156del; p.Leu81Serfs∗5 that results in the absence of exon 4 from the cDNA, frameshift, and premature stop codon. The other allele apparently harbors a donor splice site mutation c.1225 + 5G > C. This patient also has a deletion in one allele, but it is much larger than the one in our patient, which does result in frameshift and premature stop codon, but it is much earlier in the protein sequence than in our patient. The stop-codon mRNA mediated decay effect of the frameshift and chain-terminating mutations, although not proven, if present should have the same effect for those alleles, too. The heterozygous splice-site mutation is of a totally different nature than the homozygous frameshift and chain-terminating mutations in our patient. Arhan et al. [30] reported a single ARC patient with cholestasis, FS, NDI, corpus callosum agenesis, hypothyroidism, intrauterine fractures, and osteopenia, but no arthrogryposis. All 23 *VPS*33*B* exons and exon-intron boundaries were screened but no pathogenic mutations were identified. The authors postulated that pathogenic mutations might exist further into intronic regions, promoter regions, or enhancing elements, or most likely another gene is responsible for the ARC phenotype in that family. They provided no evidence that the *VIPAS*39 gene was screened for mutations. Coleman et al. [13] also reported two patients with ARC phenotype without myoskeletal deformities and thus no arthrogryposis, but they did not provide any data on the specific associated molecular gene defects.

In summary, the reported cases of ARC phenotype without arthrogryposis, thus far, are a total of four different families (five total patients) with six collective different *VPS*33*B* mutations. Reports of additional three families (three total patients) did not provide data on the respective mutations or molecular defects. Data presented in the medical and scientific literature for ARC patients without arthrogryposis are not compatible with a phenotype to genotype correlation. Various deletion, frameshift, and chain-terminating mutations, point mutations, and splice-site mutations have been encountered in the reported ARC patients without arthrogryposis. In our patient, the ARC syndrome was not clinically suspected initially. The delay in clinical diagnosis was due to the absence of arthrogryposis. Whole Exome Sequencing provided data and evidence for the presence of ARC syndrome without arthrogryposis.

Until the end of November 2019, 299 individuals with *VPS*33*B* gene and protein variants had been registered to the Leiden Open Variation Database (LOVD), with 79 of them presenting with ARC phenotype [7]. Among the registered 79 ARC patients with *VPS*33*B* mutations, 14 (17.7%) had frameshift mutations, 27 (34%) had nonsense mutations, and 33 (41.8%) were homozygous or combined heterozygous for *VPS*33*B* mutations resulting in an unknown change of the *VPS*33*B* protein. Most of ARC patients registered in the LOVD database were of Korean (*n* = 18) and Pakistani (*n* = 15) ethnicity, followed by patients of Arab (*n* = 6), South American (*n* = 6), and Turkish (*n* = 4) origin. Moreover, ARC cases caused by *VPS*33*B* mutations have been reported in 19 patients originating from 7 European ethnicities/countries [7]. The current patient is the first reported ARC patient of Greek origin with a confirmed *VPS*33*B* mutation. In our case, the homozygosity for a rare mutation implies consanguinity. However, there was no known relation between parental families, although both families originated from the same local community.

Treatment of ARC is mainly supportive consisting of ursodeoxycholic acid and fat-soluble vitamin administration, maintenance of water, acid-base, and electrolyte equilibrium, and treatment of concurrent infections. A recent study showing alterations in the gut microbiota of infants with cholestasis suggested that interventions on gut microbiota may ameliorate cholestasis-associated consequences on liver function [34]. Prognosis of ARC is invariably poor, with death occurring within the first year of life in most cases, although cases with mild phenotype and survival into adulthood have been reported [25, 28, 29]. The main causes of death include infections, hypernatremic dehydration, and bleeding complications, especially following a liver biopsy. In patients with longer lifespan, liver function may deteriorate progressively leading to cirrhosis [5]. Prognosis in the current patient was considered poor from very early on in the context of recurrent dehydration episodes and the long-term dependence on the indwelling central catheter, which is often complicated with fulminant sepsis as was the case with our patient.

## 4. Conclusions

The current ARC case adds to the spectrum of ARC-associated *VPS*33*B* mutations and provides evidence further supporting the existence of incomplete ARC phenotype. Therefore, the ARC should be suspected in all patients presented with early, low GGT cholestasis and renal tubular dysfunction, even in the absence of arthrogryposis, and relative genetic tests should be included among the first-line diagnostic tools.

## Figures and Tables

**Table 1:** Timeline.

History				
Family: unremarkable. Obstetric: mild gestational diabetes. Perinatal: unremarkable				

Age	Clinical findings	Diagnostic tests	Interventions	Diagnosis outcome

14^th^ day	Polyuria, dehydration, no malformations	Metabolic acidosis, hypernatremia, direct hyperbilirubinemia, abnormal urine excretion of electrolytes, glycosuria, increased PTL, anemia	IV fluids, electrolytes and sodium bicarbonate, feeding via an orogastric catheter	Renal tubular acidosis with Fanconi syndrome, neonatal cholestasis

44^th^ day	*Klebsiella* sepsis, persisting polyuria, growth retardation, hypotonia	As above plus aminoaciduria. Negative desmopressin test. Head US: thin corpus callosum. DNA tests ordered	As above plus antibiotics, vitamins D, E, and K, and ursodeoxycholic acid. Intravenous catheter	Incomplete ARC with nephrogenic diabetes insipidus, sepsis

3^rd^–5^th^ month	Recurrent febrile episodes and catheter-related sepsis, polyuria partly responding to hydrochlorothiazide, delayed neurodevelopment	Increasing direct bilirubin and transaminase levels cerebral MRI: hypoplastic corpus callosum Homozygosity for *VPS*33*B* mutation	As above plus hydrochlorothiazide, jejunostomy for enteral feeding	Genetically confirmed ARC syndrome

7^th^ month	Persisting *Candida albicans* sepsis, multiorgan failure	Further increasing direct bilirubin and transaminase levels	As above plus antifungal and supportive treatment	Death

**Table 2:** Blood/serum laboratory findings.

	Units	Patient values or range	Normal range
Hemoglobin	g/dl	7.3–12.9	10–14
White blood count	Cell/*μ*L	6.5–34.3	5.6–13.9
Platelet count	Cell/*μ*L	333–1135	150–450
Mean platelet volume	fL	9.4–12.2	7.5–11.0
Total bilirubin	mmol/L	68.4–342	5.0–21.0
Conjugated bilirubin	mmol/L	49.6–262	1.7–5.1
Alanine aminotransferase	IU/L	33–242	13–45
Aspartate aminotransferase	IU/L	77–444	9–80
Gamma-glutamyl transpeptidase	IU/L	23–60	15–90
Alkaline phosphatase	IU/L	334–1440	70–380
Bile acids	*μ*mol/L	115	0–8
*α*1-antitrypsin	*μ*mol/L	28	16.2–50.4
Total protein	g/L	43–57	51–71
Albumin	g/L	22–36	32–56
Urea	mmol/L	7.1–31.8	2.9–8.2
Creatinine	*μ*mol/L	45.1–87.5	18–35
Na	mmol/L	133–163	130–145
K	mmol/L	3.1–5.4	4.1–5.3
Cl	mmol/L	108–140	91–108
P	mg/dL	2.2–5.5	4.0–6.5
Ca	mmol/L	2.3–2.5	2.25–2.75
Cholesterol	mmol/L	3.8–5.3	4.4–5.2
Triglycerides	mmol/L	4.97–7.80	0.34–3.29
Lactate dehydrogenase	IU/L	208–431	<24 mo 180–430
Creatine phosphokinase	IU/L	12–39	20–200
TSH	mIU/L	1.7–12.0	<12
FT4	pmol/L	14.2–18.0	10.8–22.6

mo, month; Fl, femtolitre.

## Data Availability

Readers can obtain access to the data supporting the conclusions of the study through the corresponding author.
